# Dissect new mechanistic insights for geniposide efficacy on the hepatoprotection using multiomics approach

**DOI:** 10.18632/oncotarget.21897

**Published:** 2017-10-19

**Authors:** Shi Qiu, Aihua Zhang, Tianlei Zhang, Hui Sun, Yu Guan, Guangli Yan, Xijun Wang

**Affiliations:** ^1^ Sino-America Chinmedomics Technology Collaboration Center, National TCM Key Laboratory of Serum Pharmacochemistry, Chinmedomics Research Center of State Administration of TCM, Heilongjiang University of Chinese Medicine, Harbin, China; ^2^ Laboratory of Metabolomics, Department of Pharmaceutical Analysis, Heilongjiang University of Chinese Medicine, Harbin, China; ^3^ State Key Laboratory of Quality Research in Chinese Medicine, Macau University of Science and Technology, Avenida Wai Long, Taipa, Macau

**Keywords:** proteome, metabolome, microrna, metabolism, metabolites

## Abstract

A multi-omics approach could yield in-depth mechanistic insights. Here, we performed an integrated analysis of miRNAome, proteome and metabolome, aimed to investigate the underlying mechanism of active product geniposide in ethanol-induced apoptosis. We found that integrative meta-analysis identified 28 miRNAs, 20 proteins and 7 metabolites significantly differentially expressed, respectively. Further analysis identified geniposide extensively regulated multiple metabolism pathways and the most important related pathway was citrate cycle (TCA cycle). In addition, geniposide can improve energy metabolism benefits using the Extracellular Flux Analyzer. Of particular significance, miR-144-5p exhibits a high positive correlation with oxoglutaric acid, isocitrate dehydrogenase (IDH) 1 and 2 that involved in the TCA cycle. Furthermore,we discovered that miR-144-5p regulates TCA cycle metabolism through IDH1 and IDH2. Collectively, we describe for the first time the hepatoprotective effect of geniposide decreased miR-144-5p level, capable of regulating TCA cycle by directly targeting IDH1 and IDH2 and promoting functional consequences in cells. Integrating metabolomics, miRNAomics and proteomics approach and thereby analyzing microRNAs and proteins as well as metabolites can give valuable information about the functional regulation pattern and action mechanism of natural products.

## INTRODUCTION

Cell metabolism is regulated at multiple levels in various components e.g., g miRNAs, metabolites, proteins, and *in vivo* enzymes. The high-throughput multi-omics techniques could provide a complete picture of cell’s responses to stimulus factor [1]. Recent data suggested that miRNAs as modulators in treatment and prevention of liver fibrosis [2, 3]. Metabolomics has been widely used to explain the mechanism of drug [4]. Metabolomics has the great advantage to provide a closer link to the phenotype of a cell. Genomics gives an overview of the complete set of genetic information about phenotype [5]. A single layer of “omics” could provide limited insights into the action mechanism of drug. The integrative approach of multi-omic data may enhance the understanding of the molecular dynamics underlying the pathophysiology of diseases.

The 2015 Nobel Prize in Physiology or Medicine won by pharmacologist, Youyou Tu, was the first science Nobel awarded to a China-based scientist. Tu’s discovery was rooted in ancient Chinese herbal medicine-has brought traditional Chinese medicine (TCM) to the forefront of the global research community’s attention. Geniposide (Supplementary Figure 1), a major iridoid glycoside found in TCM *Gardenia jasminoides Ellis*, has been widely used in Asian countries. Numerous studies have demonstrated that geniposide has a protective effect for liver injury via up-regulating the expression of antioxidant enzymes [6, 7]. We previously showed that geniposide has potential hepatoprotective effect on hepatic damage rats, through regulating multiple perturbed pathways including TCA cycle, alanine, aspartate and glutamate metabolism, primary bile acid biosynthesis, etc [8, 9]. These finding highlights the potential value of geniposide as a possible treatment for hepatic injury, such as alcoholic liver disease (ALD). Recently, studies have discovered several miRNAs are aberrantly expressed after ALD. Metabolome of ALD patients led to identification of potential metabolites [10, 11].

An integrated approach that combines multi-omics data, is a very powerful tool to provide a more comprehensive understanding of biological effects of drug [12, 13]. Furthermore, integration of proteome and metabolome are directly interconnected as protein levels influence the metabolic profile of a cell system [14, 15]. Although each layer of the omic profile allows a comprehensive survey for that particular type of disease associations, the cross talk between multiple molecular layers cannot be easily assessed. In this context, we performed an integrated analysis of the miRNAome, proteome and metabolome profiling and mapped the coordinated systemic responses in a cell model of ethanol-induced apoptosis in normal mice hepatocytes (Enmh) treatment with geniposide. Taken altogether, we provide a paradigmatic example of integrating association analysis approach to map the crosstalk among the miRNAome, proteome and metabolome, and permits searching for new drug targets.

## RESULTS

### Hepatoprotective effects

Treatment with geniposide effectively prevented the loss of cell viability (Supplementary Figure 2A, 2B). We next evaluated whether geniposide affects Enmh cells apoptosis. Geniposide significantly decreased the number of apoptotic cells compared to model group (Supplementary Figure 2C). In contrast, the geniposide promoted a significant cell cycle arrest at G1 phase compared to the model group (Supplementary Figure 2D). Taken together, it suggested that geniposide ameliorates the ethanol-mediated injury in Enmh cells. However, molecular details for the hepatoprotective functions need further explore.

### Global microRNA profiling

**Figure 1:**
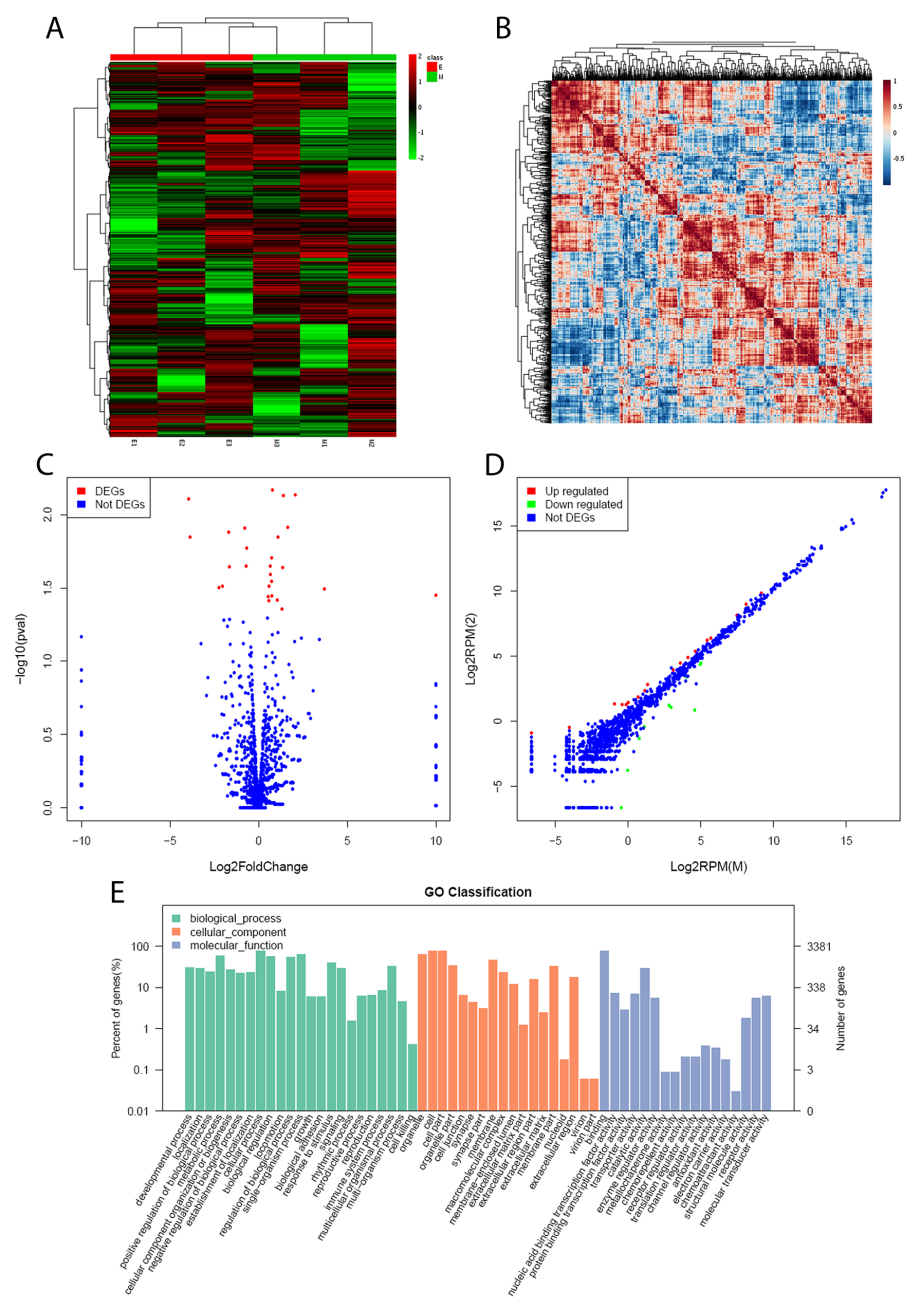
miRNA expression profile **(A)** Heat map of microRNA expression; within the heatmap, red color represents higher levels of relative activity/expression; black represents intermediate levels, and green represents lower levels of relative activity/expression; **(B)** comparative expression of the highly expressed miRNAs using Heatmapfunction in R package; **(C)** volcano plot for the screened the differential expression of miRNA; **(D)** the differential expression of host miRNAs as a function; **(E)** GO annotation on host targets of the miRNAs.

We first investigated the influence of geniposide impact on the microRNAome in the Enmh cells. Unsupervised hierarchical clustering of the microRNAome data from the cellular samples showed the differentially expressed abundance and clear differentiation between the geniposide and Enmh groups (Figure 1A). A total of 1695 miRNAs were detected, and of which 28 miRNAs were significantly changed during geniposide treatment (Figure 1B). Comparative expression of the highly expressed miRNAs was shown in Figure 1C. Heatmap plot of top expressed miRNAs was shown in Supplementary Figure 3. 18 miRNAs were upregulated, whereas 10 miRNAs were downregulated in the Enmh group (Figure 1D, Supplementary Table 1). We predicted the mRNA targets of each of the altered miRNAs. The basic biological function of each putative target gene was classified based on the Gene Ontology enrichment analysis. Results revealed that these host targets of miRNAs were associated with the biological regulation, metabolic pathway, signaling process, stimulus response, and immune response (Figure 1E).

### Proteomic analyses

**Figure 2:**
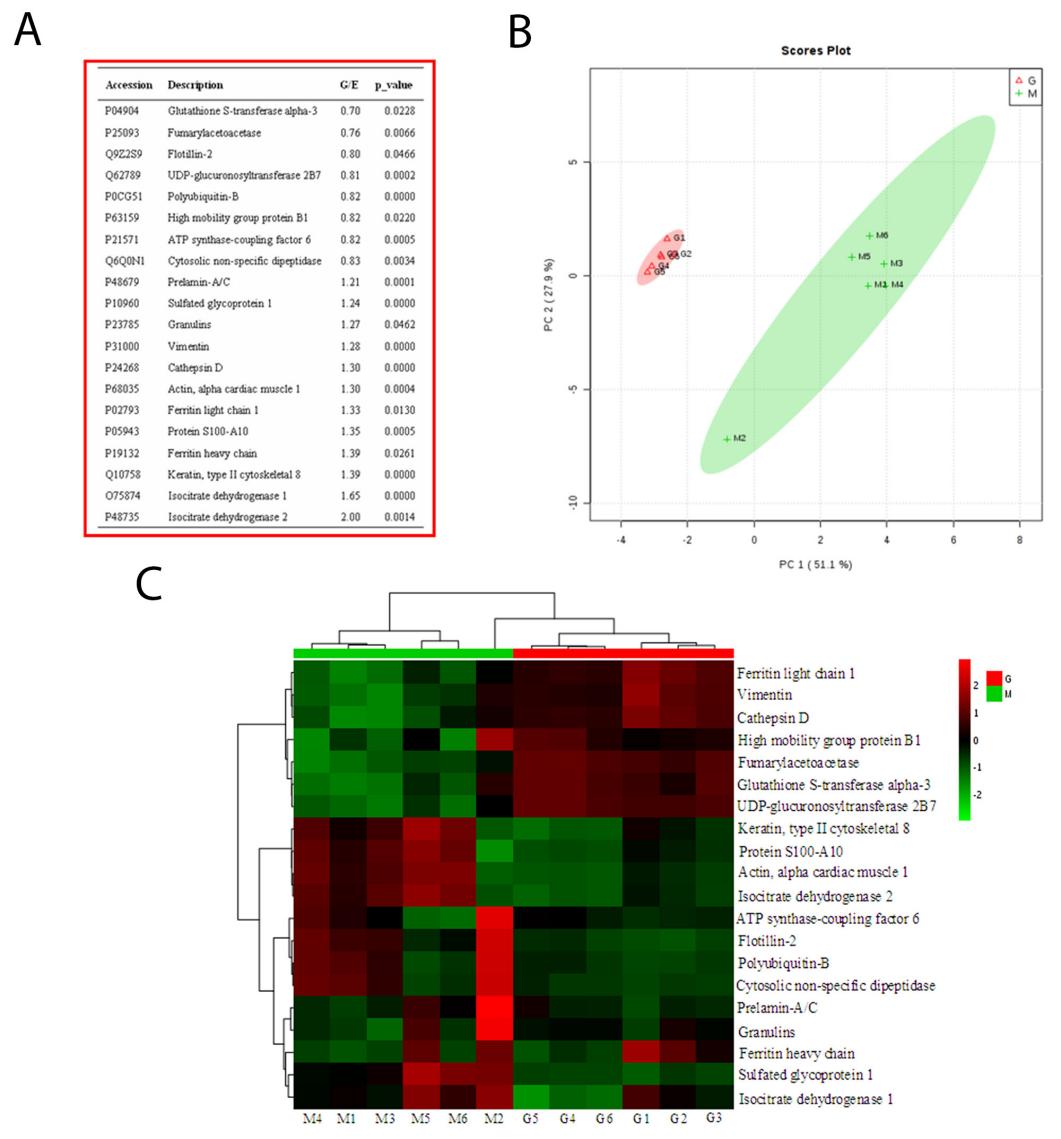
Proteomic profile **(A)** List of the differentially expressed proteins; **(B)** Unsupervised PCA analysis of the most variable proteins; **(C)** Heatmap for the protein expression which significantly differentiate two clusters.

iTRAQ labeling was combined with an LC-MS/MS experiment to identify differentially expressed proteins (DEPs). As a result, 1069 proteins (FDR<1%) were identified. Among these proteins, the expression levels of 20 proteins were markedly altered in geniposide group (P < 0.05; Supplementary Table 2) compared to Enmh groups. Proteomic analyses results showed 12 proteins that up-regulated and 8 others that down-regulated among the detected proteins (Figure 2A). Principal components analysis (Figure 2B) and hierarchical clustering analysis (Figure 2C) of DEPs data showed clear segregation between the geniposide and Enmh groups. Comparative expression of the highly DEPs was shown in Supplementary Figure 4. GO analyses indicated enrichment in DEPs associated with many important pathways such as ‘‘immune response’’, “regulation of cellular process” and ‘‘RNA metabolic process’’ which play a core role for geniposide therapeutics (Supplementary Figure 5).

### Metabolic profile analyses

**Figure 3:**
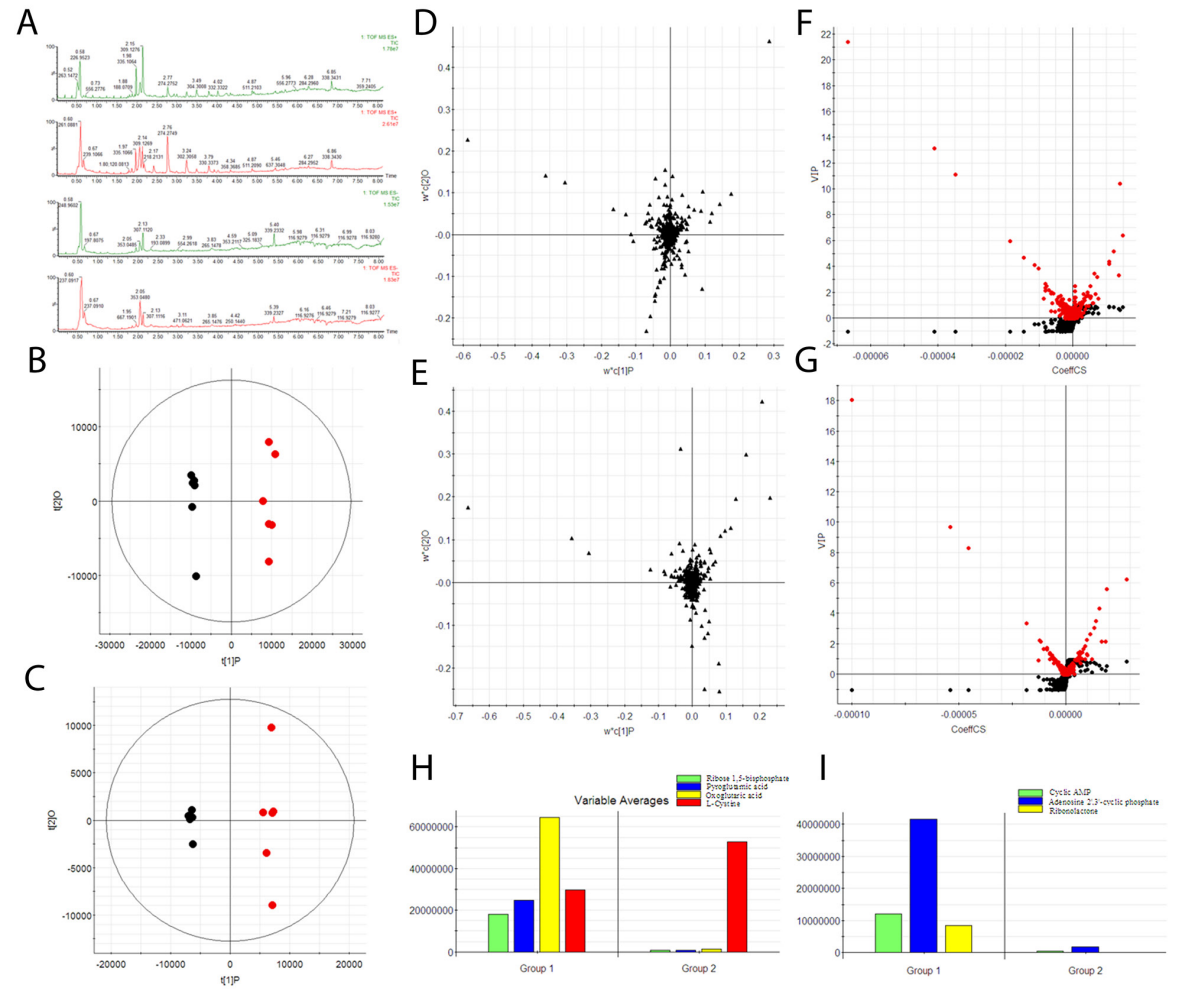
Cellular metabolome characterization **(A)** Chromatogram of UPLC-MS; **(B)** principal component analysis score plot classifying the geniposide (black) and model group (red) in positive ion mode; **(C)** principal component analysis score plot classifying the geniposide (black) and model group (red) in negative ion mode; **(D)** loading plot of PLS-DA model of LC-MS spectra data from intracellular metabolites in positive ion mode; **(E)** loading plot of PLS-DA model of LC-MS spectra data from intracellular metabolites in negative ion mode; **(F)** VIP-plot of OPLS-DA model of LC-MS spectra data from intracellular metabolites in positive ion mode; **(G)** VIP-plot of OPLS-DA model of LC-MS spectra data from intracellular metabolites in negative ion mode; **(H)** histogram of significantly differentially detected metabolites in positive ion mode; **(I)** histogram of significantly differentially detected metabolites in negative ion mode.

The representative total ion chromatograms were presented in Figure 3A. Low molecular mass metabolites could be separated well in the short time of 8 min. Using a high-throughput and untargeted UPLC-MS screen, as described in MS data analysis, a total of 2964 ion peaks from intracellular data were obtained. Scores plot of OPLS-DA showed clear separation between the control and model groups in ESI^+^ (Figure 3B) and ESI^-^ (Figure 3C). From the loading-plot of PLS-DA, the ions furthest away from the origin maybe regarded as the differentiating metabolites (Figure 3D, 3E). Variables were identified according to a threshold of VIP (variable importance in the projection) (Figure 3F, 3G). Following the criterion above (VIP>8), nine significantly changed metabolites which are linked to geniposide were identified in intracellular metabolome. Expression levels of significantly differential metabolites were showed in Figure 3H, 3I. Geniposide had a significant impact on metabolic profiling and 7 metabolites such as oxoglutaric acid and cyclic AMP etc., were found significantly differentially expressed (Supplementary Table 3). Then, two metabolisms were filtered out as potential pathway for the geniposide, and the most important pathway is the TCA cycle (Supplementary Figure 6 and Supplementary Table 4). Results also manifest that geniposide could change the abnormal energy metabolism status, because the TCA cycle is fundamental for mitochondrial energy production.

### Geniposide improved energy metabolism

**Figure 4:**
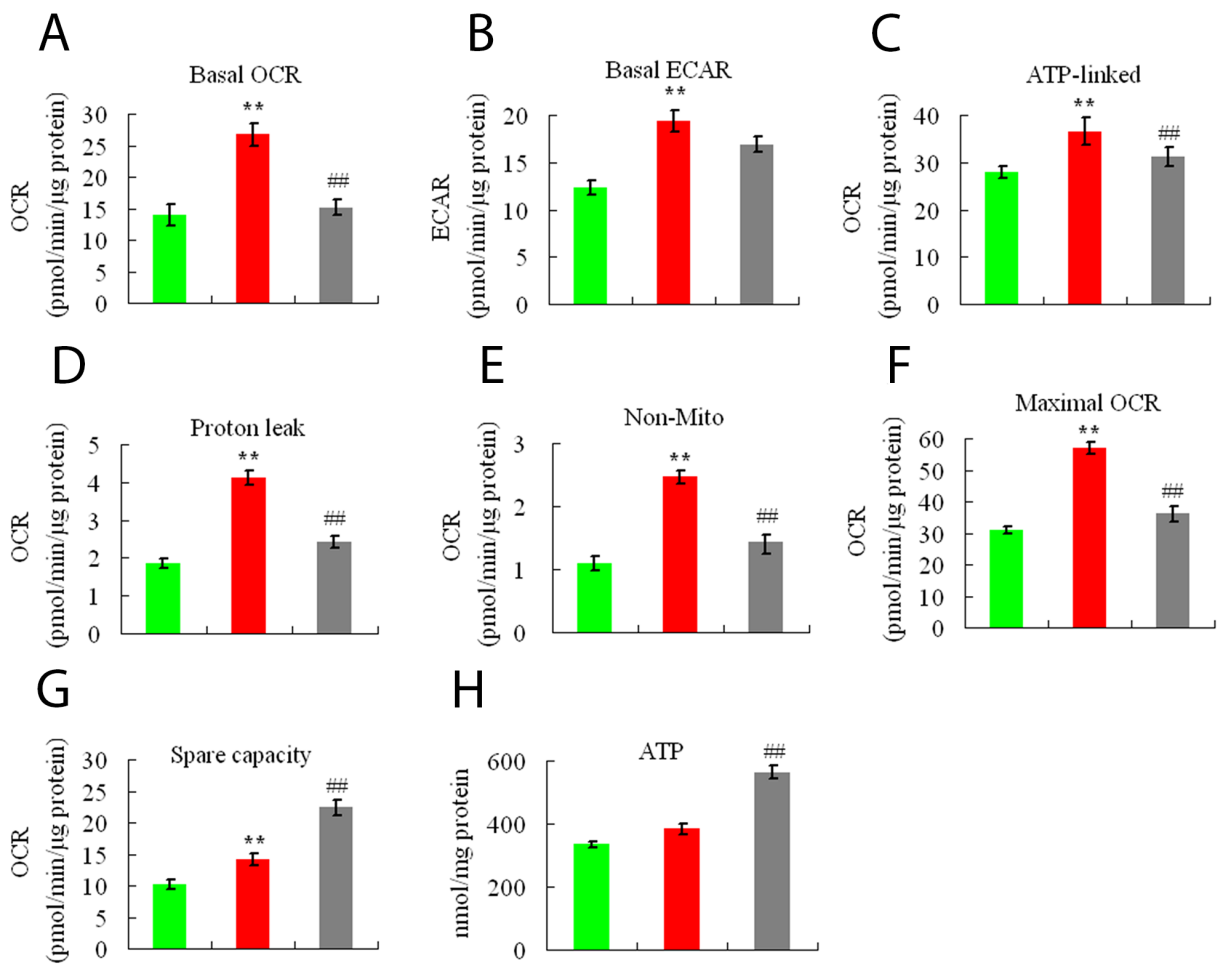
Geniposide affects energy metabolism on Enmh cells **(A)** Basal OCRs; **(B)** basal ECARs; **(C)** ATP-linked OCRs; **(D)** proton leak; **(E)** nonmitochondrial OCRs (non-mito); **(F)** maximal OCRs; **(G)** spare capacity; **(H)** The cellular ATP level. Results are mean ± SEM, and are representative of three independent experiments. ^**^ p<0.01 vs. for 24 h control group; ^##^ p<0.01 vs. for 24 h ethanol group.

We next tested whether geniposide could modulate the energy metabolism. As shown in Figure 4A, 4B, compared with the control group, ethanol treatment markedly increased basal cellular ECARs and OCRs, respectively. Ethanol increased in the absolute level of the ATP-linked respiration, proton leak, and non-mitochondrial oxygen consumption (Figure 4C-4E). Ethanol treatment also increased the mitochondrial respiratory (Figure 4F), and reduced the ATP production (Figure 4H). We next tested the regulative effects of geniposide on the OCR and ECAR of Enmh cells. We found that when the cells were cotreated with ethanol and geniposide for 24 h, the OCRs was reduced (Figure 4A). No decrease in ECARs was found in Enmh cells treated by geniposide (Figure 4C). However, Enmh cells cotreated with geniposide for 24 h were markedly downregulated (Figure 4D, 4E). We found that Enmh cells treated with geniposide could upregulate the mitochondrial respiratory capacities (Figure 4F, 4G). Furthermore, geniposide treatment significantly increased ATP content than that in the model group (Figure 4H).

### Merged network with multi-omics characteristics

**Figure 5:**
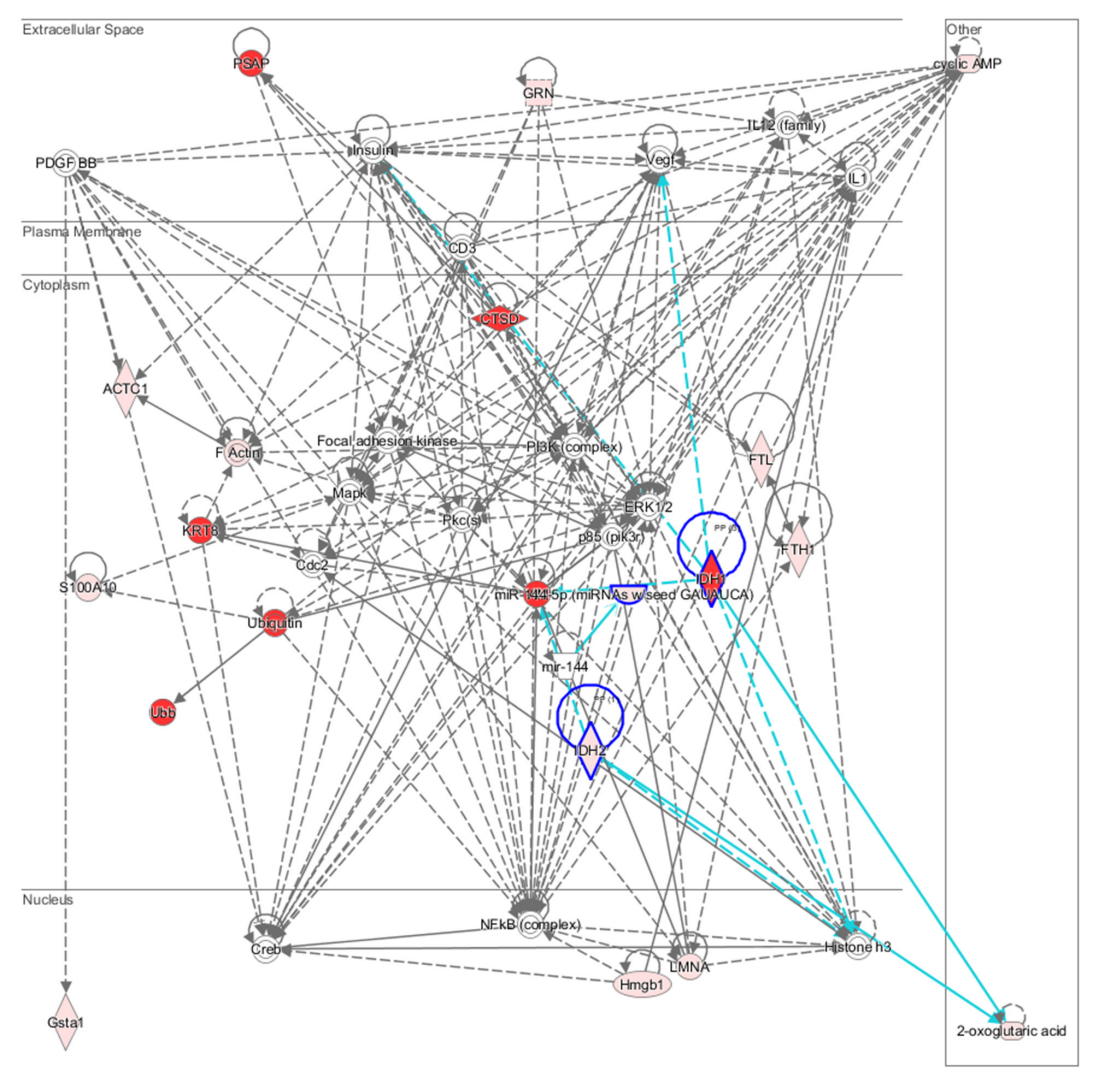
Synthesized molecular networks The networks are obtained by analyzing the differentially expressed biomolecules (listed in Tables S1, S2 and S3) using Ingenuity IPA. Green nodes; upregulated biomolecule expression, red nodes; downregulated biomolecule expression.

The combination and interpretation of complex data for all the miRNAome, proteome and metabolome was performed via IPA software. Figure 5 showed molecular pathways affected by geniposide exposure, and highlights the identified up-regulated and down-regulated miRNAs, proteins and metabolites and their interconnection in the network. It reported the most significant molecular networks affected in response to geniposide treatment, data were obtained by analyzing the differentially expressed miRNA, proteins and metabolites. Results demonstrate that the major networks altered in response to geniposide were related to cell interaction and signaling, cell assembly and organization, cellular proliferation and growth.

### Correlation analysis of differentially expressed moleculars

We next investigated the correlations among the miRNAome, proteome and metabolome. MicroRNA level was correlated with proteins and metabolites analysis by PCMS software (2015SR164324, Harbin, China) using pearson’s correlation. A PCMS correlation model from multi-omics data sets was established to discover correlation. According to the correlation coefficient (r ≥ 0.9), a total of 25 statistically significant (p-values ≤ 0.05) associations have been identified (Supplementary Figure 7). A number of miRNAs were strongly correlated with the significantly changed proteins and metabolites. PCMS analysis showed that the top three miRNAs (mmu-miR-96-5p, mmu-miR-221-5p, mmu-miR-144-5p) had an extremely relationship with proteins and metabolite, might play important roles in the hepatoprotective effect of geniposide. Upregulated mmu-miR-96-5p and mmu-miR-221-5p demonstrated a broad correlation with the intracellular metabolite profiles. Most importantly, mmu-miR-144-5p exhibits a high positive correlation with oxoglutaric acid, isocitrate dehydrogenase 1 (IDH1) and isocitrate dehydrogenase (IDH2) that involved in the TCA cycle.

### MiR-144-5p regulates TCA cycle through IDH1 and IDH2

In our analysis model, miR-144-5p showed the high correlations to metabolites. So, we focused on exploring the functions of miR-144-5p on the TCA cycle metabolism. Of note, two metabolic enzymes, IDH 1 and 2, were identified involved from the TCA cycle.To exlpore the effect of miR-144-5p on IDH2 and IDH1, the overexpressed the miRNA were transiently in Aml12 cells, and repressed mRNA (Supplementary Figure 8A) and protein (Supplementary Figure 8B). It also inhibited the activity of IDH1 and IDH2 (Supplementary Figure 8C), indicating that specific inhibition.

## DISCUSSION

Zhizi is one of the natural medicinal plants for treatment of liver disorders and geniposide is one of the active ingredients in Zhizi, and shows therapeutic effects [8]. However, the molecular mechanism of the hepatoprotective effect remains largely unknown. A lot of studies have been carried out on anti-oxidant, and anti-inflammatory activities [18]. A better understanding of the mechanisms regulating hepatic cell injury may lead to the effective approaches for liver diseases. Therefore, we next examined the effects of geniposide against ethanol-induced hepatic injury.

Proteomics and metabolomics, combined with miRNAome, are excellent tools to screen the effect mechanisms [19, 20]. Cross-linking different techniques can improve the knowledge and understanding in liver injury as shown in this work. Firstly, geniposide significantly decreased the number of apoptotic cells compared to model group, suggesting that geniposide generates hepatoprotective effects. Global microRNA profiling shows clear differentiation of the geniposide and Enmh groups. A total of most highly differentially expressed 28 miRNAs were significantly changed during geniposide treatment, 18 of which were upregulated, and 10 miRNAs were downregulated. And then, the expression levels of 20 proteins were identified and significantly altered in geniposide group compared to Enmh cells, using iTRAQ labeling combined with an LC-MS/MS. Principal components analysis showed distinct segregation between the geniposide and Enmh groups. Metabolite profile analysesshowed that geniposide had a significant impact on the metabolic profiling and found 7 significantly differentially expressed metabolites. The major networks altered in response to geniposide were related to cell interaction and signaling, cell organization, cellular proliferation and growth. Pathway analysis suggested that geniposide treatment mainly regulated TCA cycle metabolism. The results also manifest that geniposide could change the energy metabolism, because the TCA cycle is fundamental for mitochondrial energy production. Energy metabolism disorder is a key causal factor of liver damage [21–25]. In this study, we also found that geniposide markedly increased the spare respiratory and capacity cellular ATP conten of cells exposed to the ethanol condition.

A PCMS correlation model from multi-omics data sets was established to discover correlation. According to the correlation coefficient (r ≥ 0.9) of PCMS correlation model, a total of 25 statistically significant (p-values ≤ 0.05) associations have been identified. We found that miR-144-5p exhibits a high positive correlation with oxoglutaric acid, IDH1 and 2 involved in TCA cycle. We found that MiR-144-5p regulates TCA cycle through IDH1 and IDH2. Overall, as elucidated in Supplementary Figure 8D, hepatoprotective effect of geniposide modulated MiR-144-5p affecting TCA cycle through IDH1 and IDH2.

In this study, our integrative multi-omics analysis not only enables the discovery of new targets. We found that multi-omics identified 28 miRNAs, 20 proteins and 7 metabolites significantly differentially expressed, respectively. Interesting, miR-144-5p that affect TCA cycle through IDH1 and IDH2 was identified as a key regulator of geniposide treating hepatic injury. Metabolites produced by the cells are potentially important physiological modulators and miRNAs have been identified as promoters or suppressors of disease progression. Some reports have demonstrated that mutations in *IDH1* and *IDH2* are recurrent in brain tumors [26, 27]. In summary, we highlighted the emerging roles of miR-144-5p may serve as a potentially pharmacological target of geniposide for therapeutic intervention in hepatic injury.

In this work, we present a multi-omics approach to gain a better understanding of the cellular effects after geniposide exposure *in vitro*. This analysis provided mechanistic insights into how geniposide regulated the miRNA, metabolite and protein levels. Key regulators were identified from the close association among multi-omics. Further analysis identified geniposide could improve metabolism benefits by regulating TCA cycle pathway. Interesting, miR-144-5p exhibits a high correlation with oxoglutaric acid, IDH1 and IDH2 that involved in the TCA cycle. Furthermore,we discovered that geniposide could decrease miR-144-5p level, directly targeting IDH1 and IDH2 and promoting functional recovery in Enmh cells by regulating the TCA cycle. Taken altogether, we provide a paradigmatic example of integrating association analysis method to map the crosstalk among the miRNAome, and proteome as well as metabolome, offering opportunities to understand valuable information about functional regulation pattern and action mechanism of natural products, and permits searching for new drug targets to enhance drug development.

## MATERIALS AND METHODS

### Chemicals

Geniposide (Purity: > 98%, Supplementary Figure 1) was acquired from the NIPBP (Beijing, China), and then dissolved in 0.1% dimethyl sulfoxide (DMSO). All the other reagents not mentioned were of the highest grade commercially available.

### Cell culture

Primary hepatocytes were isolated from 6-week-old ICR male mice by two-step collagenase perfusion [16]. The harvested hepatocytes were seeded in 24 wells-plates adding per well 1 mL cell suspension containing 1×10^5^ cells/mL medium. The next day, the medium was supplemented with ethanol (50 mM) for 24-h culture. After 24 h, the medium was washed and the wash solution discarded, and geniposide dissolved in DMSO to obtain the final concentrations of 0.9 ug/ml. All cells were kept in a 37°C incubator with 5% CO_2_. The cells were washed with cold PBS 3 times, and then stored in-80 °C. In each experiment, untreated geniposide cells served as control group.

### Cell cycle and apoptosis analysis

Cells were collected by trypsinization, washed 3 times with PBS, fixed in 70% ethanol followed by staining with 20 μg/ml propidium iodide. The stained cells were analyzed by flow cytometry (Merck-Millipore, USA). Apoptosis was analyzed by flow cytometry (Merck-Millipore, USA). The data were analyzed using Guava software 2.6 (Merck-Millipore, USA) for Flow Cytometry.

### MiRNA profiling and analysis

MicroRNA profiling was performed using miRCURY LNA microRNA arrays (Exiqon, Denmark). The hybridization was performed the standard protocols. Microarray slide was scanned with a DNA microarray scanner, after hybridization. Feature extraction software v10 was used to convert the scanned images into TXT files, which were imported in R software for quality control. Next, these intensities were normalized.

### Proteome profiling by mass spectrometry

Protein fractionation was carried out in accordance with manufacturer’s protocol. Strong-cation exchange and reversed phase liquid chromatography (AB SCIEX, CA, USA) and LC-MS/MS analysis were performed. For iTRAQ based quantitative proteomics experiments, the digested peptides were labelled according to the protocol. The sample spots were analyzed by MALDI TOF/TOF (AB SCIEX, CA, USA).

### Metabolic profiling analyses

#### Metabolite extraction

For intracellular metabolites analysis, the cells were washed twice with PBS and then extracted with 80% cold methanol. Pellets were extracted as mentioned above with cold methanol and cold water (50:50) and subjected to three freeze-thaw cycles. The supernatants were centrifuged at 4°C for 7 min. Supernatant was lyophilized after removal of methanol.

#### UPLC-MS

The gradient mobile phase was composed of acetonitrile containing 0.1% formic acid and water with 0.1% formic acid. The column temperature was kept at 45°C. The flow rate was 0.4 mL/min and injection volume was 2μL. Nitrogen was used as the collision gas. MS spectra was acquired on QTF/MS equipped with an ESI^+^ and ESI^-^ electrospray ionization source. The parameters are as follows: source temperature 110 °C, cone voltage 20 V, capillary voltage 3000 V, desolvation temperature 350°C, and desolvation gas flow 600 L/h. Leucine enkaphalin was used as the reference compound for accurate mass measurement.

#### Data collection and analysis

Data was further analyzed by Markerlynx software (Waters Corp., Milford, USA) for alignment, peak finding, peak integration and Rt correction. The normalized data were then used for multivariate analysis, such as principal components analysis (PCA), and orthogonal projection to latent structure discriminant analysis (OPLS-DA). Pathways analysis were performed with Metaboanalyst [17] based on KEGG database.

### Ingenuity pathway analysis

To identify biological pathways significantly overrepresented, pathway enrichment analysis was performed by IPA software which consists of functions, pathways and network models. The pathways and functional categories with FDR < 0.05 were regarded as significantly associated.

### Measurement of energy metabolism

Oxygen consumption rates (OCRs) and extracellular acidification rates (ECARs) in cells were measured by an Extracellular Flux Analyzer (Seahorse Bioscience, Billerica, MA). The instrument measures the extracellular flux changes of oxygen and protons in the medium surrounding the cells seeded in XF96-well plates. To test the effect of geniposide on the OCR and ECAR, cells were seeded in XF96-well plates and incubated for 24 h. The cells were then switched to DMEM 1 h prior to the beginning of the ATP assay.

### Quantitative RT-PCR analysis

Quantitative RT-PCR was performed by QuantiTect SYBR Green RT-PCR kit (QIAGEN). Primer sequences were obtained from Eurogentec. The 2^−ΔΔCT^ method was used to compute the relative amounts of target mRNA in the samples,.

### Immunoblotting

Proteins were isolated and quantified using a BCA assay (Pierce, UK). Anti-β-actin, Anti-IDH1 and -IDH2 antibody was purchased from Sigma-Aldrich. Proteinlysates were resolved in 10% acrylamide gels and their relative intensities were determined using the Image J software (Bethesda, MD , USA).

### Statistical analysis

Metabolomics patterns were visualized using Ezinfo 2.0 software. Significant differences in microRNA profiles were visually evaluated to detect outliers using R. P < 0.05 was considered significant. We performed a consensus clustering of cell samples on the basis of the results from miRNA, protein and metabolites consensus clustering analyses. MicroRNA levels were correlated with metabolite levels analysis by PCMS software (2015SR164324, Harbin, China). To determine correlation analysis from multi-omics data sets, the correlation coefficient (r) described the degree of correlation.

## SUPPLEMENTARY MATERIALS FIGURES AND TABLES



## Abbreviations

TCM: Traditional Chinese medicine
ALD: alcoholic liver disease
Enmh: ethanol-induced apoptosis in normal mice hepatocytes
IDH1: isocitrate dehydrogenase 1
IDH2: isocitrate dehydrogenase
PCA: principal components analysis
OPLS-DA: orthogonal projection to latent structure discriminant analysis
OCRs: oxygen consumption rates
ECARs: extracellular acidification rates
