# Supplementary material for: Dissect new mechanistic insights for geniposide efficacy on the hepatoprotection using multiomics approach
Source: Oncotarget. 2017 Oct 19;8(65):108760–70. doi: 10.18632/oncotarget.21897 (PMC5752478; doi:10.18632/oncotarget.21897)
Supplement: Supplementary file 1 [file oncotarget-08-108760-s001.pdf]

Dissect new mechanistic insights for geniposide efficacy using multiomics approach

SUPPLEMENTARY MATERIALS

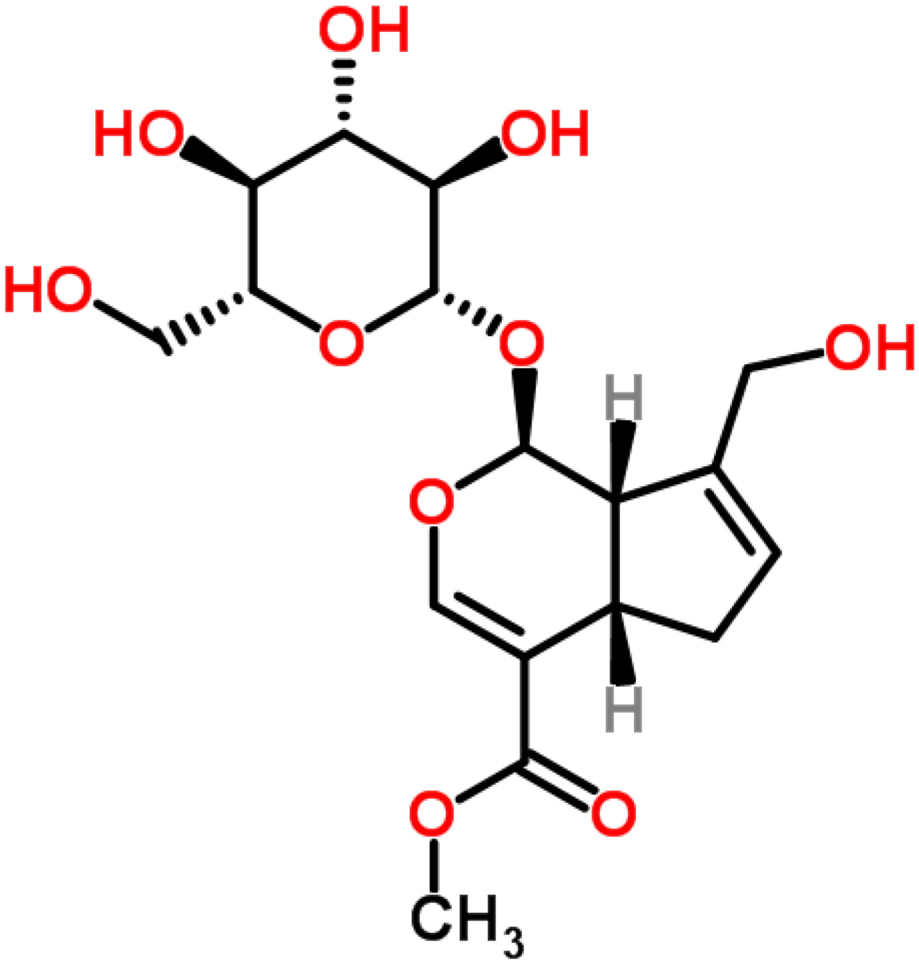

Supplementary Figure 1: The chemical structure of geniposide generated using ChemSpider.

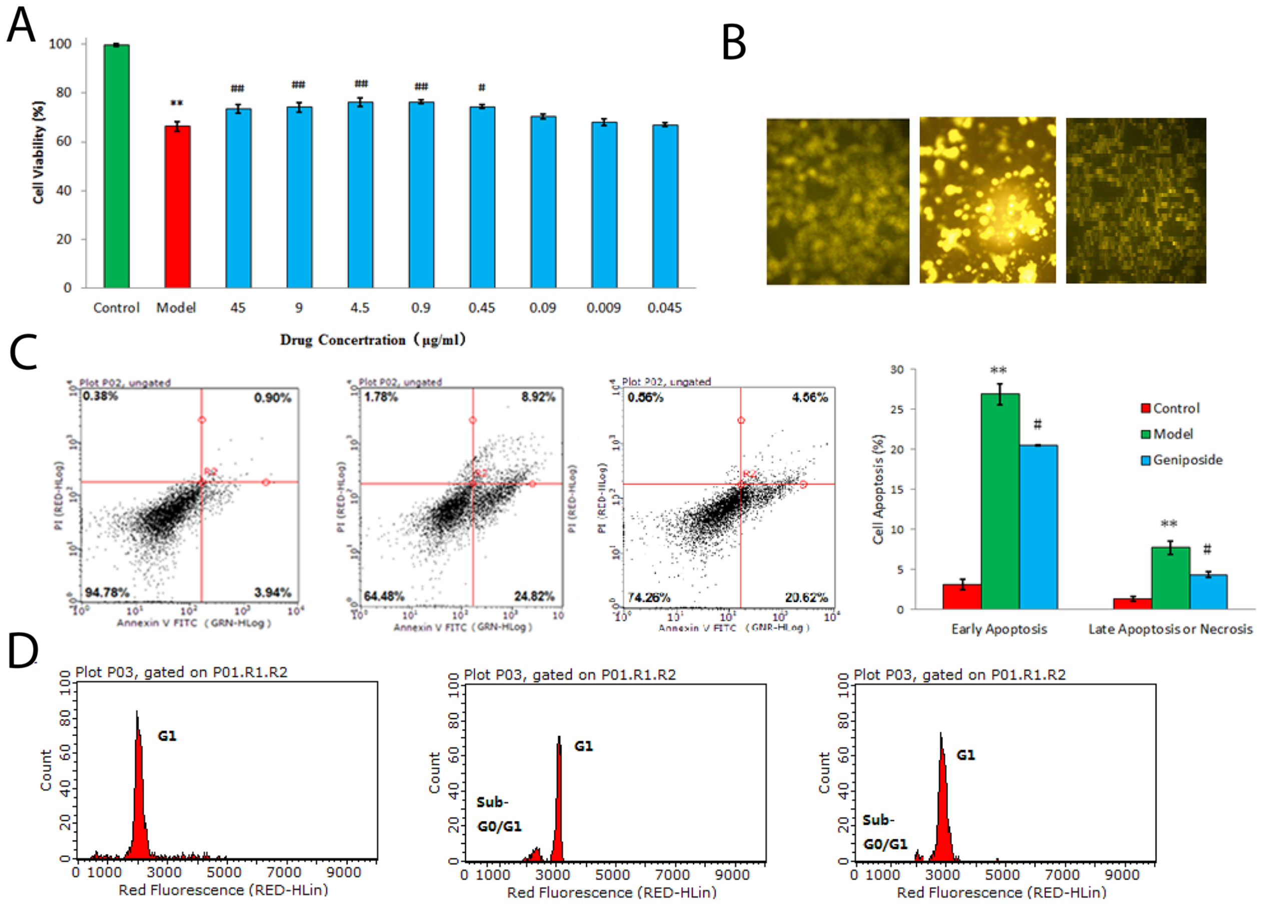

Supplementary Figure 2: Geniposide generates hepatoprotective effects on Enmh cells. **(A)** Enmh cells Viability under the different concentrations of geniposide; **(B)** Fluorescence analysis of geniposide on Enmh cells; **(C)** Apoptosis analysis of geniposide on Enmh cells by flow cytometry; **(D)**, Geniposide promoted a cell cycle arrest at G1 phase compared to the model and analysed by flow cytometry.

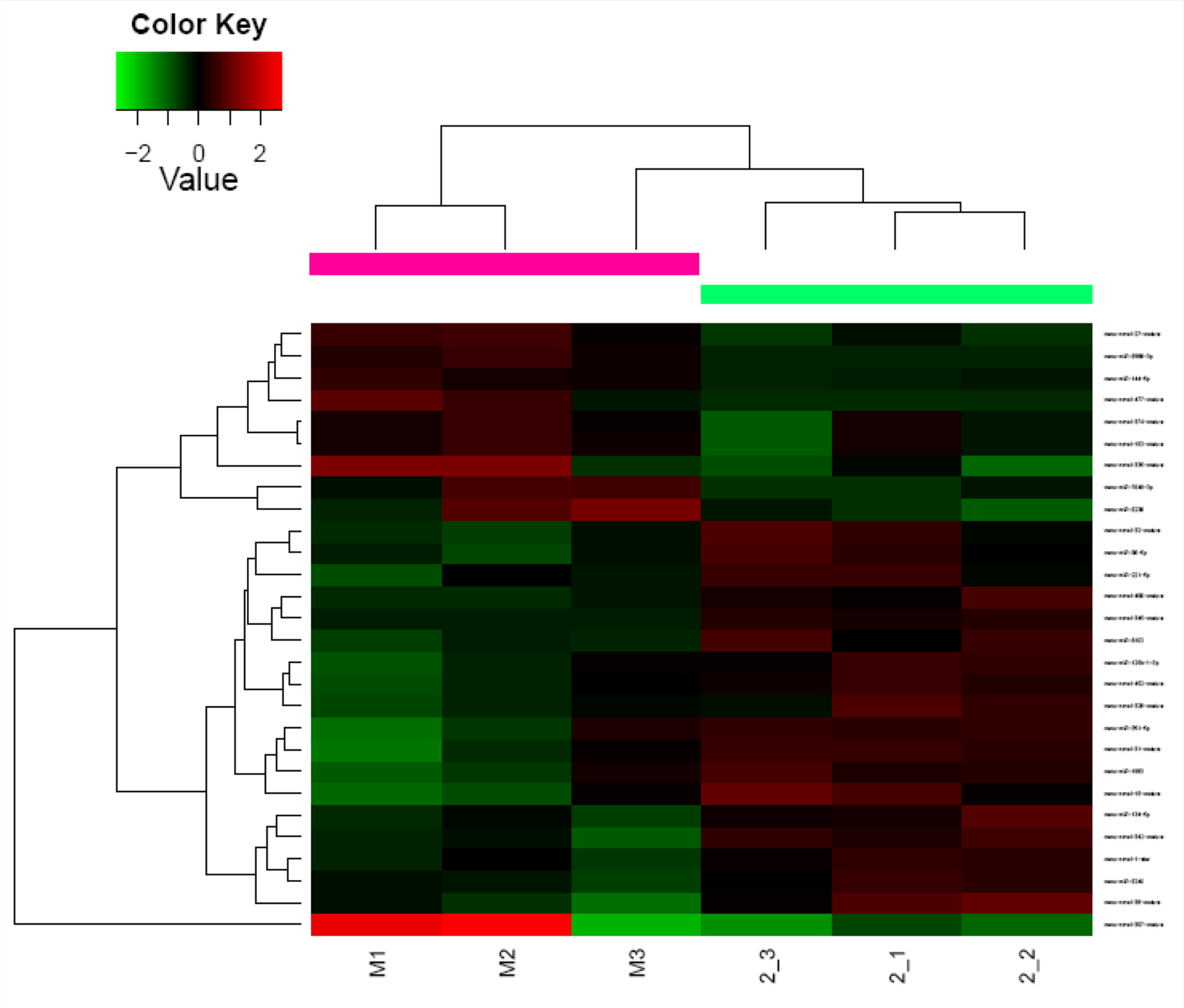

Supplementary Figure 3: Heatmap of the top differentially expressed miRNAs.

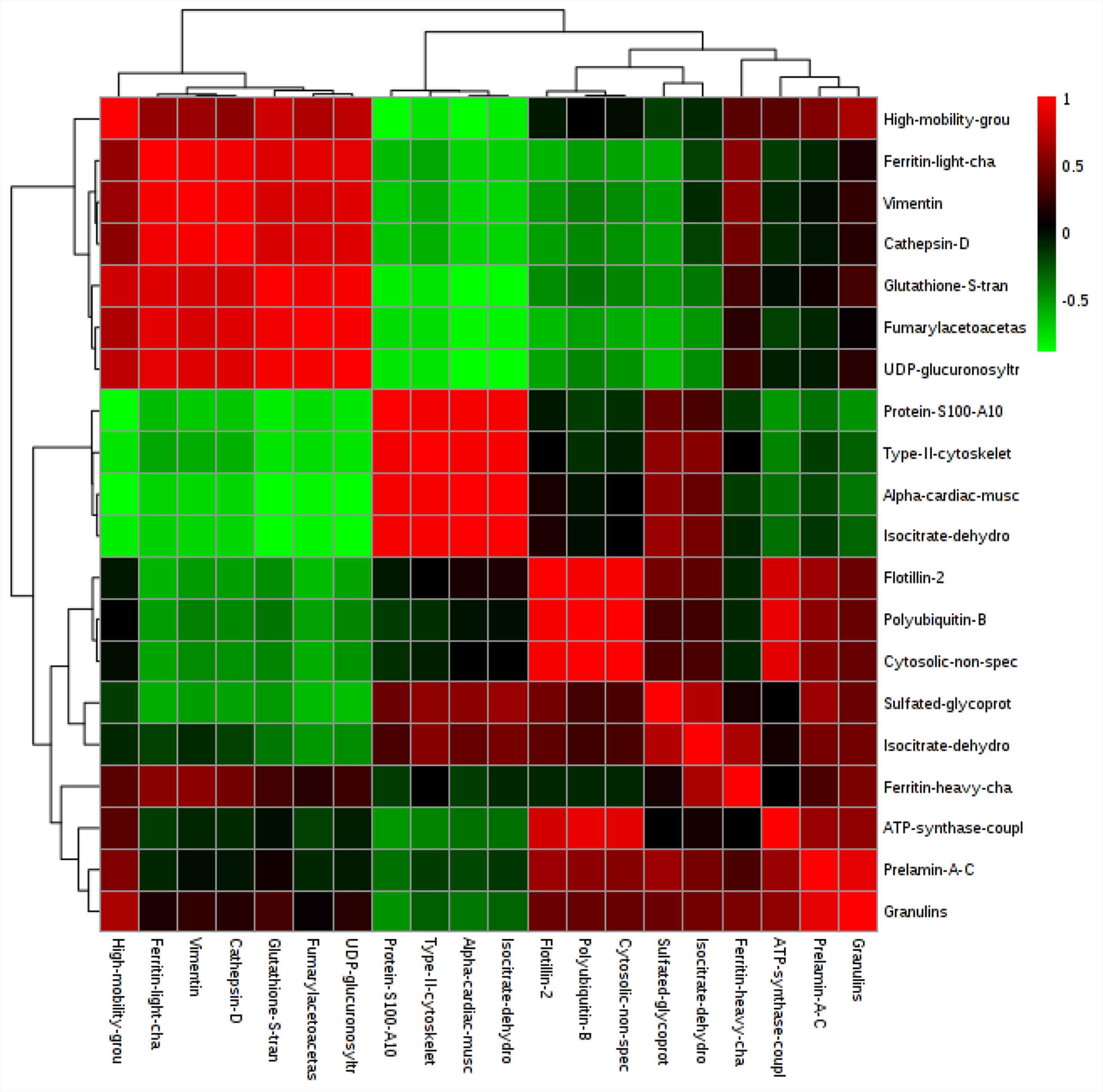

Supplementary Figure 4: Comparative expression of the highly expressed proteins using Heatmap function in R package.

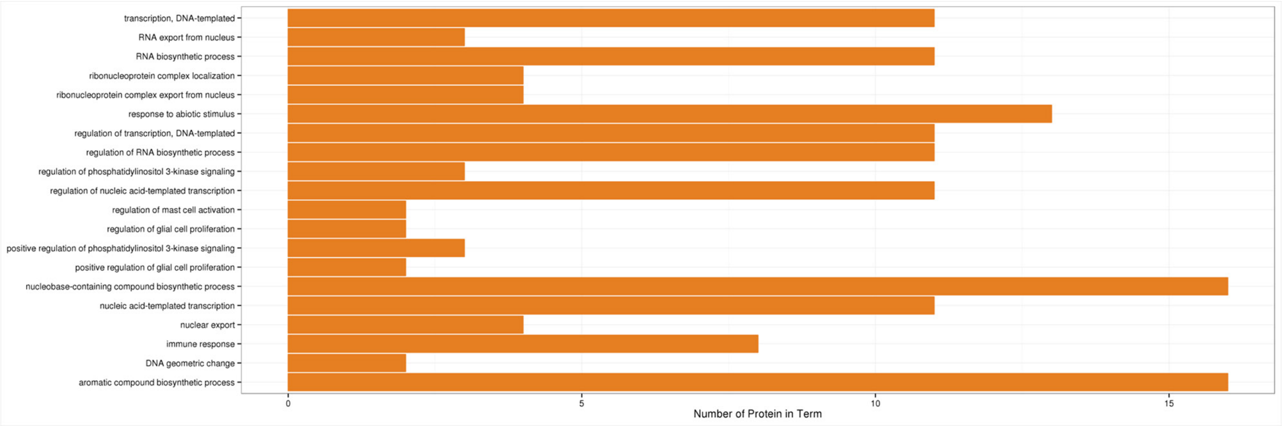

Supplementary Figure 5: GO term enrichment of the differentially expressed proteins.

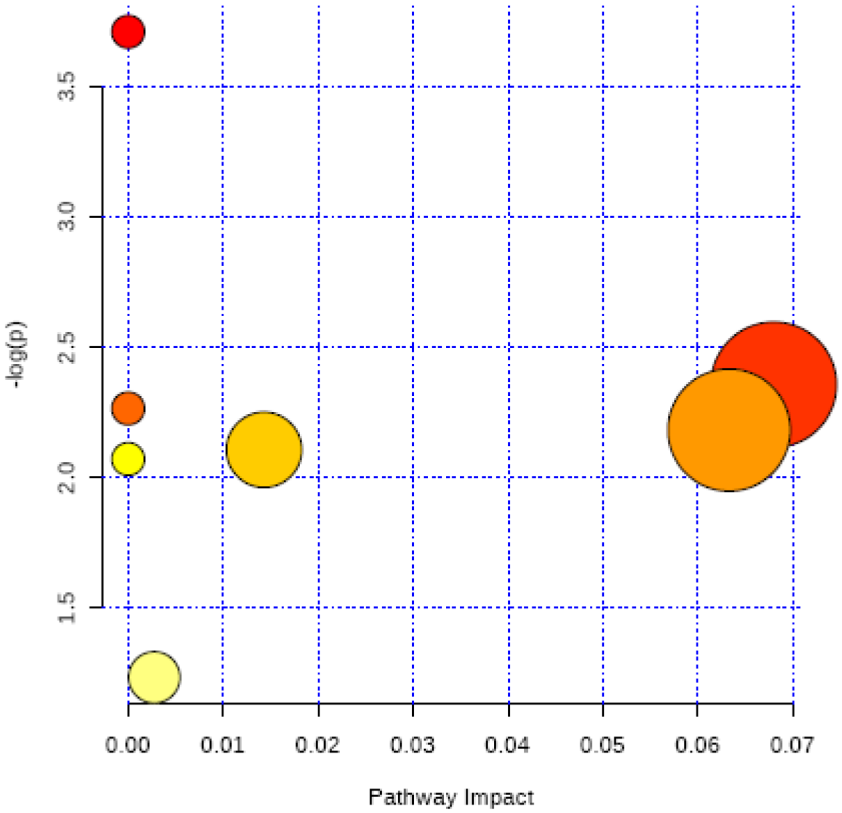

Supplementary Figure 6: Summary of pathway analysis with MetaboAnalyst 2.0 on intracellular metabolites.

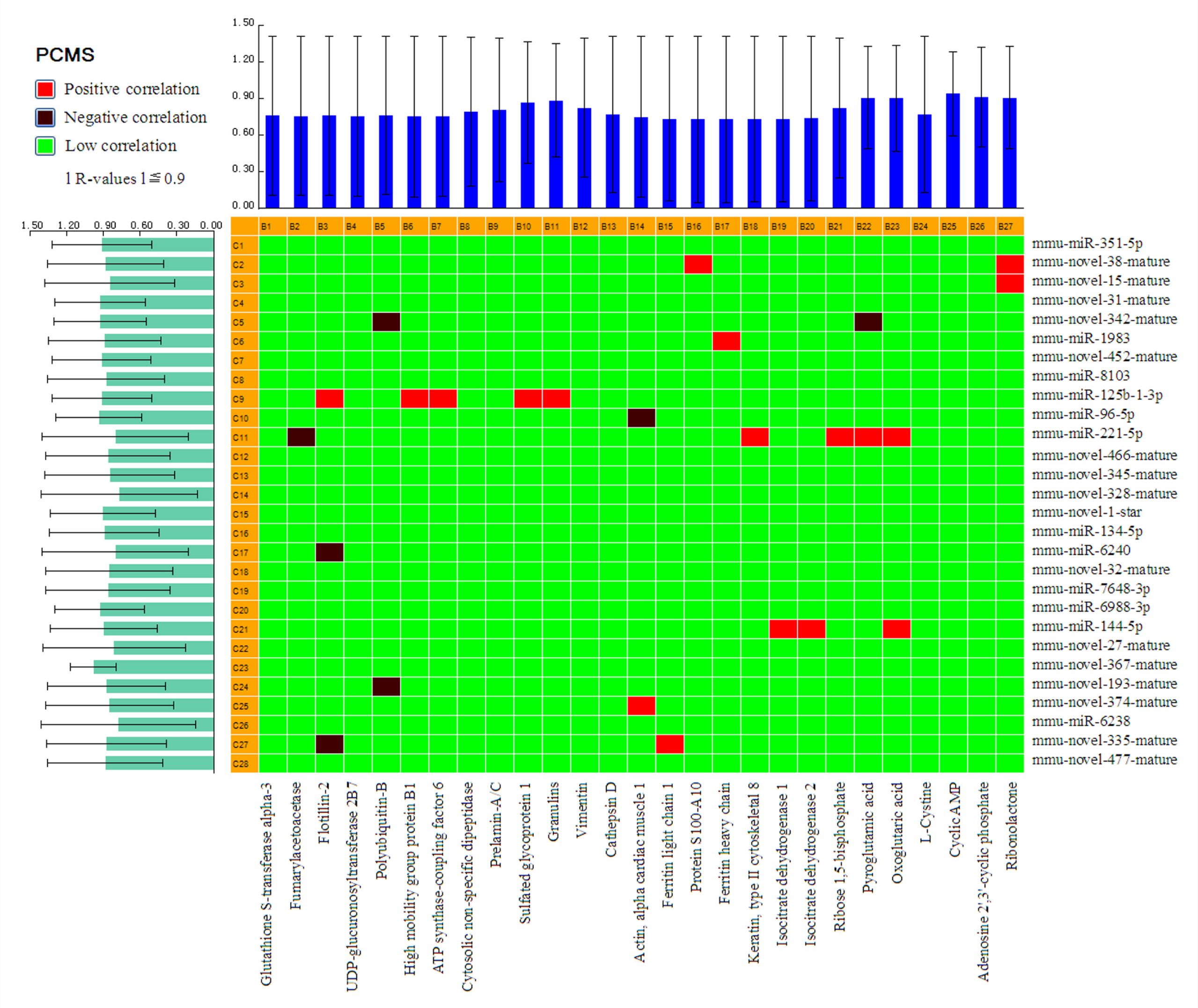

Supplementary Figure 7: Correlation module of multi-omics data The heat map is generated from the PCMS via Pearson correlation coefficient values between normalized microRNA expression and differentially expressed protein and metabolite levels. The horizontal axis shows all detectable microRNAs, whereas the vertical axis exhibits altered protein and metabolites. The color bars are shown in red (upregulated) and black (downregulated), respectively. See Supplementary Tables S1, S2 and S3 for a list of genes, proteins and metabolites.

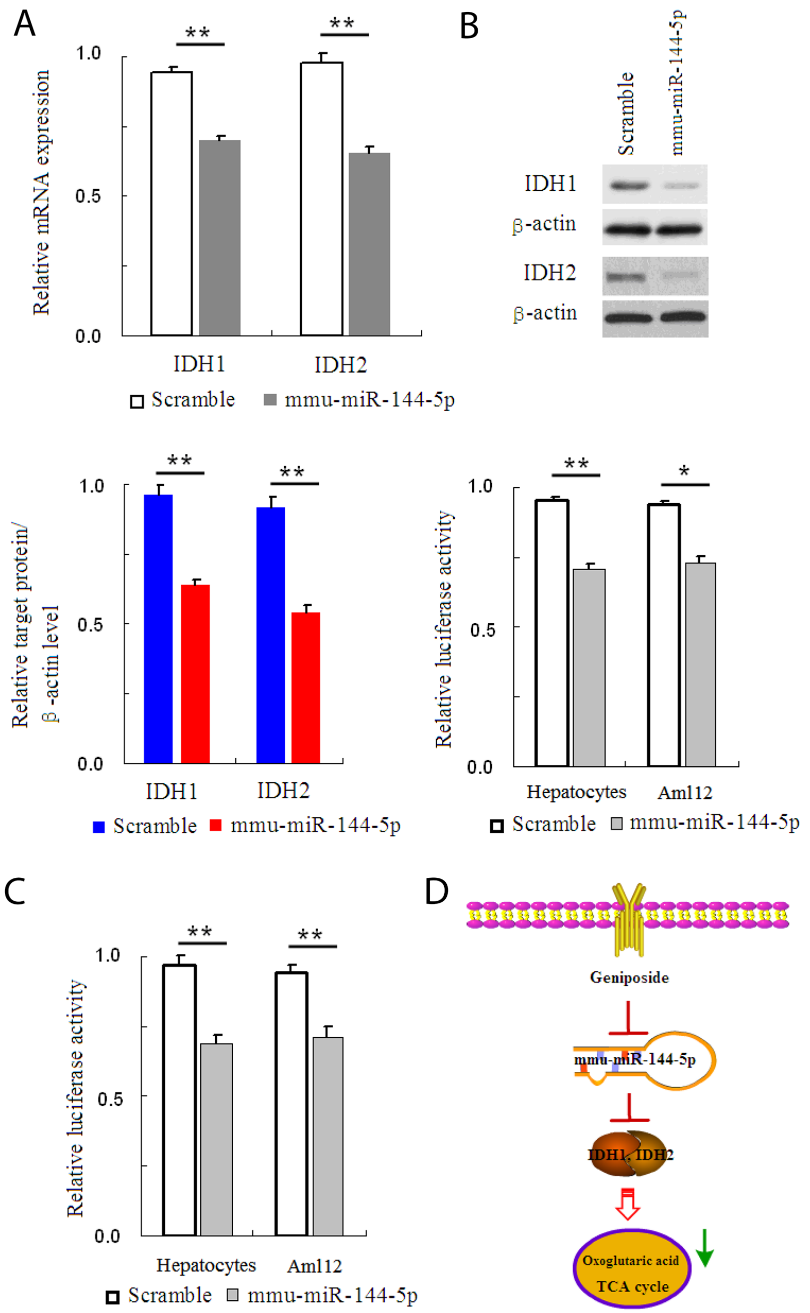

Supplementary Figure 8: MiR-144-5p regulates TCA cycle through targeting IDH1 and IDH2. **(A)** Quantitative reverse transcriptase–PCR detection of IDH1 and IDH2 mRNA in Aml12 cells transfected with either miR-144-5p mimic or a negative mimic control (scramble). Data are normalized to β-actin and shown as mean±s.e.m.,n=3. *P<0.05; **(B)** Western blotting for DH1 and IDH2 in Aml12 transfected with miR-144-5p mimic or scramble control. A representative immune blot and a bar chart of relative protein levels are shown (mean ± SEM, n=3). **(C)** Validation of direct targeting of DH1 and IDH2 by miR-144-5p using a luciferase reporter assay; **(D)** Graphical overview of the mechanism underlying geniposide-regulated.

Supplementary Table 1: Summary of the differentially expressed miRNA

No	Type	id	baseMean	baseMeanA	baseMeanB	foldChange	log2FoldChange	Pval
1	Up	mmu-miR-351-5p	2124.2504	1557.8552	2690.6455	1.73	0.7884	0.0068
2	Up	mmu-novel-38-mature	8.0973	3.0984	13.0963	4.23	2.0796	0.0074
3	Up	mmu-novel-15-mature	25.4461	14.1206	36.7717	2.60	1.3808	0.0074
4	Up	mmu-novel-31-mature	8.4674	4.0917	12.8430	3.14	1.6502	0.0123
5	Up	mmu-novel-342-mature	19.8451	12.6754	27.0148	2.13	1.0917	0.0142
6	Up	mmu-miR-1983	319.7646	241.3672	398.1620	1.65	0.7221	0.0197
7	Up	mmu-novel-452-mature	177.7216	138.4835	216.9596	1.57	0.6477	0.0225
8	Up	mmu-miR-8103	10.0289	5.6671	14.3906	2.54	1.3444	0.0230
9	Up	mmu-miR-125b-1-3p	125.9470	97.6355	154.2586	1.58	0.6599	0.0257
10	Up	mmu-miR-96-5p	64.9921	49.0092	80.9750	1.65	0.7244	0.0286
11	Up	mmu-miR-221-5p	361.8778	290.0703	433.6854	1.50	0.5802	0.0307
12	Up	mmu-novel-466-mature	2.0639	0.2929	3.8350	13.09	3.7109	0.0321
13	Up	mmu-novel-345-mature	1.3846	0.0000	2.7691	Inf	Inf	0.0355
14	Up	mmu-novel-328-mature	90.8605	68.1477	113.5733	1.67	0.7369	0.0359
15	Up	mmu-novel-1-star	1231.8954	1009.6930	1454.0977	1.44	0.5262	0.0364
16	Up	mmu-miR-134-5p	14.2200	9.2068	19.2333	2.09	1.0628	0.0382
17	Up	mmu-miR-6240	4087.8957	3301.3163	4874.4750	1.48	0.5622	0.0389
18	Up	mmu-novel-32-mature	8.9306	5.1546	12.7066	2.47	1.3016	0.0444
19	Down	mmu-miR-7648-3p	2.9097	5.4641	0.3553	0.07	-3.9429	0.0079
20	Down	mmu-miR-6988-3p	2.0658	4.1317	0.0000	0.00	Inf	0.0083
21	Down	mmu-miR-144-5p	74.2397	93.8074	54.6719	0.58	-0.7789	0.0123
22	Down	mmu-novel-27-mature	8.2028	12.5269	3.8787	0.31	-1.6914	0.0132
23	Down	mmu-novel-367-mature	74.3526	139.1422	9.5630	0.07	-3.8630	0.0143
24	Down	mmu-novel-193-mature	148.5869	183.5081	113.6657	0.62	-0.6910	0.0170
25	Down	mmu-novel-374-mature	142.9965	176.9603	109.0327	0.62	-0.6987	0.0226
26	Down	mmu-miR-6238	25.6675	38.9641	12.3708	0.32	-1.6552	0.0229
27	Down	mmu-novel-335-mature	28.0576	45.1751	10.9401	0.24	-2.0459	0.0310
28	Down	mmu-novel-477-mature	5.9930	9.8933	2.0927	0.21	-2.2411	0.0317

Supplementary Table 2: Identified the regulated proteins

No	Accession	Description	# PSMs	# AAs	MW [kDa]	calc. pI	G/E	p_value
1	P04904	Glutathione S-transferase alpha-3	3	221	25.3	8.75	0.70	0.0228
2	P25093	Fumarylacetoacetase	5	419	45.9	7.17	0.76	0.0066
3	Q9Z2S9	Flotillin-2	5	428	47	5.20	0.80	0.0466
4	Q62789	UDP-glucuronosyltransferase 2B7	33	530	60	8.27	0.81	0.0002
5	P0CG51	Polyubiquitin-B	68	305	34.3	7.53	0.82	0.0000
6	P63159	High mobility group protein B1	4	215	24.9	5.74	0.82	0.0220
7	P21571	ATP synthase-coupling factor 6	8	108	12.5	9.44	0.82	0.0005
8	Q6Q0N1	Cytosolic non-specific dipeptidase	7	475	52.7	5.66	0.83	0.0034
9	P48679	Prelamin-A/C	42	665	74.3	6.98	1.21	0.0001
10	P10960	Sulfated glycoprotein 1	18	554	61.1	5.25	1.24	0.0000
11	P23785	Granulins	3	588	63.3	6.47	1.27	0.0462
12	P31000	Vimentin	71	466	53.7	5.12	1.28	0.0000
13	P24268	Cathepsin D	15	407	44.7	7.09	1.30	0.0000
14	P68035	Actin, alpha cardiac muscle 1	233	377	42	5.39	1.30	0.0004
15	P02793	Ferritin light chain 1	13	183	20.7	6.43	1.33	0.0130
16	P05943	Protein S100-A10	8	95	11.1	6.77	1.35	0.0005
17	P19132	Ferritin heavy chain	6	182	21.1	6.30	1.39	0.0261
18	Q10758	Keratin, type II cytoskeletal 8	190	483	54	6.00	1.39	0.0000
19	O75874	Isocitrate dehydrogenase 1	122	452	50.9	8.69	1.65	0.0000
20	P48735	Isocitrate dehydrogenase 2	30	414	46.7	6.99	2.00	0.0014

Supplementary Table 3: List of significantly differential metabolites

No	Retention time (min)	m/z	Compound ID	Adducts	Formula	Mass Error (ppm)	Description	Anova (p)	VIP
1	0.93	310.9894	HMDB11688	M+H	C5H12O11P2	-1.83	Ribose 1,5-bisphosphate	0.0001	11.14
2	1.61	130.0502	HMDB00267	M+H	C5H7NO3	2.67	Pyroglutamic acid	0.0001	13.18
3	3.14	147.0271	HMDB00208	M+H	C5H6O5	-1.72	Oxoglutaric acid	0.0004	21.40
4	2.62	241.0323	HMDB00192	M+H	C6H12N2O4S2	2.02	L-Cystine	0.0006	10.45
5	2.15	328.0437	HMDB00058	M-H	C10H12N5O6P	-1.66	Cyclic AMP	0.0004	9.73
6	4.21	330.0596	HMDB11616	M+H	C10H12N5O6P	-0.56	Adenosine 2',3'-cyclic phosphate	0.0026	18.07
7	1.71	147.0292	HMDB01900	M-H	C5H8O5	-0.78	Ribonolactone	0.0008	8.32

Supplementary Table 4: Pathway analysis with MetPA on intracellular metabolites

Name	Total	Expected	Hits	Raw p	Impact
D-Glutamine and D-glutamate metabolism	5	0.0247	1	0.024491	0
Citrate cycle (TCA cycle)	20	0.0988	1	0.094906	0.06799
Butanoate metabolism	22	0.10868	1	0.10396	0
Alanine, aspartate and glutamate metabolism	24	0.11856	1	0.11293	0.06329
Glutathione metabolism	26	0.12844	1	0.12183	0.01431
Cysteine and methionine metabolism	27	0.13338	1	0.12625	0
Purine metabolism	68	0.33592	1	0.29178	0.00275

Note: Total is the total number of compounds in the pathway; the Hits is the actually matched number from the user uploaded data; the Impact is the pathway impact value calculated from pathway topology analysis.
